# 3,4,7-Trimethyl-2-(4-methyl­phen­yl)-2*H*-pyrazolo­[3,4-*d*]pyridazin-5-ium thio­cyanate

**DOI:** 10.1107/S160053681004883X

**Published:** 2010-11-27

**Authors:** Hatem A. Abdel-Aziz, Ahmed Bari, Seik Weng Ng

**Affiliations:** a Department of Pharmaceutical Chemistry, College of Pharmacy, King Saud University, Riyadh 11451, Saudi Arabia; b Department of Chemistry, University of Malaya, 50603 Kuala Lumpur, Malaysia

## Abstract

1,1′-[5-Methyl-1-(4-methyl­phen­yl)-1*H*-pyrazole-3,4-di­yl)di­ethan­one condenses with thio­semicarbazide in the presence of acetic acid to form the title salt, C_15_H_17_N_4_^+^·NCS^−^. The fused-ring system of the cation is almost planar (r.m.s. deviation = 0.020 Å) and the aromatic substituent is aligned at an angle of 48.2 (1)° with respect to the mean plane of the fused-ring system. The N atom at the 5-position is protonated and forms a N—H⋯N hydrogen bond to the thio­cyanate cointer-ion.

## Related literature

For reviews on pyrazolo-pyridazines, see: Akbas & Berber (2006 ▶); Matiichuk *et al.* (2009 ▶). For a related structure, see: Dinçer *et al.* (2004 ▶).
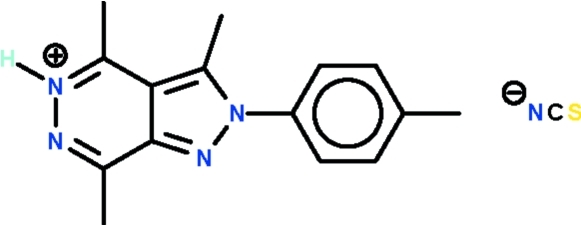


## Experimental

### 

#### Crystal data

C_15_H_17_N_4_^+^·NCS^−^
*M* *_r_* = 311.41
Monoclinic, 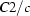

*a* = 18.2445 (5) Å
*b* = 7.3341 (2) Å
*c* = 24.2396 (8) Å
β = 106.121 (3)°
*V* = 3115.89 (16) Å^3^
*Z* = 8
Mo *K*α radiation
μ = 0.21 mm^−1^
*T* = 100 K
0.30 × 0.10 × 0.05 mm

#### Data collection

Agilent SuperNova diffractometer
Absorption correction: multi-scan (*CrysAlis PRO*; Agilent Technologies, 2010 ▶) *T*_min_ = 0.939, *T*_max_ = 0.990
7809 measured reflections
3491 independent reflections
2968 reflections with *I* > 2σ(*I*)
*R*_int_ = 0.026

#### Refinement

*R*[*F* ^2^ > 2σ(*F* ^2^)] = 0.035
*wR*(*F* ^2^) = 0.096
*S* = 1.00
3491 reflections
207 parameters
H atoms treated by a mixture of independent and constrained refinement
Δρ_max_ = 0.32 e Å^−3^
Δρ_min_ = −0.27 e Å^−3^

### 

Data collection: *CrysAlis PRO* (Agilent Technologies, 2010 ▶); cell refinement: *CrysAlis PRO*; data reduction: *CrysAlis PRO*; program(s) used to solve structure: *SHELXS97* (Sheldrick, 2008 ▶); program(s) used to refine structure: *SHELXL97* (Sheldrick, 2008 ▶); molecular graphics: *X-SEED* (Barbour, 2001 ▶); software used to prepare material for publication: *publCIF* (Westrip, 2010 ▶).

## Supplementary Material

Crystal structure: contains datablocks global, I. DOI: 10.1107/S160053681004883X/bt5414sup1.cif

Structure factors: contains datablocks I. DOI: 10.1107/S160053681004883X/bt5414Isup2.hkl

Additional supplementary materials:  crystallographic information; 3D view; checkCIF report

## Figures and Tables

**Table 1:** Hydrogen-bond geometry (Å, °)

*D*—H⋯*A*	*D*—H	H⋯*A*	*D*⋯*A*	*D*—H⋯*A*
N1—H1⋯N5	0.91 (2)	1.86 (2)	2.7668 (18)	175.6 (19)

**Fig. 1.**
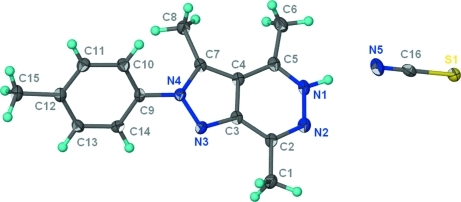
Anisotropic displacement ellipsoid plot (Barbour, 2001) of C15H17N4+ NCS– the 70% probability level; hydrogen atoms are drawn as spheres of arbitrary radius.

**Fig. 2.**
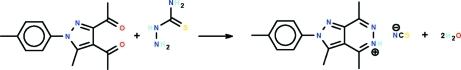
Preparation of the title compound.

## supplementary crystallographic information

### Comment

As part of our studies on the pharmaceutical applications of pyrazole derivatives, we had intended to synthesis the thiosemicarbazide condensation product of the pyrazole-dialdeyde, 1,1'-[5-methyl-1-(4-tolyl)-1*H*-pyrazol-3,4-diyl)diethanone, but the reaction yielded instead a pyrazolo[3,4-*d*]pyridazine (Scheme I, Fig. 1). The reaction was probably catalyzed by acetic acid; the reaction involves a cyclization followed by the formation of the thiocyanate ion (Fig. 2). The fused-ring system of the cation is planar (r.m.s. deviation 0.020 Å), and the aromatic substituent is aligned at 48.2 (1) ° with respect to the mean plane of the fused-ring. The nitrogen atom at the 5-position is protonated; the carbon–nitrogen double-bond involving the protonated nitrogen atom is somewhat longer [1.323 (2) Å] than the carbon–nitrogen double-bond involving the unprotonated one [1.310 (2) Å]. As the protonated nitrogen atom forms a hydrogen bond to the nitrogen end of the anion, the negative charge of the anion probably resides on this atom.

Pyrazolo[3,4-*d*]pyridazines are more conveniently synthesized directly, from the reaction of 1*H*-pyrazole-3-carboxylic acids and hydrazines (Akbas & Berber, 2006). A review of their molecular design is given by Matiichuk *et al.* (2009). For the crystal structure of a related compound, see: Dinçer *et al.* (2004).

### Experimental

1,1'-[5-Methyl-1-(4-tolyl)-1*H*-pyrazol-3,4-diyl)diethanone (0.26 g, 1 mmol) and thiosemicarbazide (0.18 g, 2 mmol) were heated in an ethanol/water (1/1, 50 ml) mixture for 4 h; acetic acid (0.5 ml) was added. The solid that separated on cooling was collected and recrystallized from ethanol to yield yellow prisms.

### Refinement

Carbon-bound H-atoms were placed in calculated positions (C–H 0.95–0.98 Å) and were included in the refinement in the riding model approximation, with *U*_iso_(H) set to 1.2–1.5 times *U*_eq_(C).

The amino H-atom was located in a difference Fourier map and was freely refined.

### Crystal data

**Table:**

C_15_H_17_N_4_^+^·NCS^−^	*F*(000) = 1312
*M**_r_* = 311.41	*D*_x_ = 1.328 Mg m^−^^3^
Monoclinic, *C*2/*c*	Mo *K*α radiation, λ = 0.71073 Å
Hall symbol: -C 2yc	Cell parameters from 4295 reflections
*a* = 18.2445 (5) Å	θ = 2.2–29.3°
*b* = 7.3341 (2) Å	µ = 0.21 mm^−^^1^
*c* = 24.2396 (8) Å	*T* = 100 K
β = 106.121 (3)°	Prism, yellow
*V* = 3115.89 (16) Å^3^	0.30 × 0.10 × 0.05 mm
*Z* = 8	

### Data collection

**Table:**

Agilent SuperNova diffractometer	3491 independent reflections
Radiation source: fine-focus sealed tube	2968 reflections with *I* > 2σ(*I*)
graphite	*R*_int_ = 0.026
Detector resolution: 10.4041 pixels mm^-1^	θ_max_ = 27.5°, θ_min_ = 2.3°
ω scans	*h* = −16→23
Absorption correction: multi-scan (*CrysAlis PRO*; Agilent Technologies, 2010)	*k* = −9→9
*T*_min_ = 0.939, *T*_max_ = 0.990	*l* = −31→23
7809 measured reflections	

### Refinement

**Table:**

Refinement on *F*^2^	Primary atom site location: structure-invariant direct methods
Least-squares matrix: full	Secondary atom site location: difference Fourier map
*R*[*F*^2^ > 2σ(*F*^2^)] = 0.035	Hydrogen site location: inferred from neighbouring sites
*wR*(*F*^2^) = 0.096	H atoms treated by a mixture of independent and constrained refinement
*S* = 1.00	*w* = 1/[σ^2^(*F*_o_^2^) + (0.0472*P*)^2^ + 3.0958*P*] where *P* = (*F*_o_^2^ + 2*F*_c_^2^)/3
3491 reflections	(Δ/σ)_max_ = 0.001
207 parameters	Δρ_max_ = 0.32 e Å^−^^3^
0 restraints	Δρ_min_ = −0.27 e Å^−^^3^

### Fractional atomic coordinates and isotropic or equivalent isotropic displacement parameters (Å^2^)

**Table:**

	*x*	*y*	*z*	*U*_iso_*/*U*_eq_	
S1	0.41884 (2)	1.03962 (5)	0.678227 (15)	0.01918 (11)	
N1	0.30697 (7)	0.87179 (17)	0.44978 (5)	0.0175 (3)	
N2	0.37098 (7)	0.79621 (17)	0.43977 (5)	0.0182 (3)	
N3	0.28626 (6)	0.74548 (16)	0.28642 (5)	0.0148 (2)	
N4	0.21174 (6)	0.79970 (16)	0.26392 (5)	0.0142 (2)	
N5	0.33646 (8)	0.96211 (19)	0.56469 (6)	0.0255 (3)	
C1	0.43819 (8)	0.6806 (2)	0.37464 (6)	0.0195 (3)	
H1A	0.4780	0.6633	0.4109	0.029*	
H1B	0.4564	0.7659	0.3502	0.029*	
H1C	0.4262	0.5632	0.3549	0.029*	
C2	0.36843 (8)	0.75572 (19)	0.38665 (6)	0.0156 (3)	
C3	0.29986 (8)	0.78638 (18)	0.34177 (6)	0.0146 (3)	
C4	0.23596 (8)	0.86541 (18)	0.35465 (6)	0.0146 (3)	
C5	0.24134 (8)	0.91093 (19)	0.41184 (6)	0.0164 (3)	
C6	0.18028 (9)	0.9977 (2)	0.43299 (7)	0.0221 (3)	
H6A	0.1980	1.0103	0.4749	0.033*	
H6B	0.1344	0.9214	0.4226	0.033*	
H6C	0.1684	1.1185	0.4155	0.033*	
C7	0.17924 (8)	0.87516 (19)	0.30233 (6)	0.0147 (3)	
C8	0.10123 (8)	0.9549 (2)	0.28774 (6)	0.0193 (3)	
H8A	0.0845	0.9835	0.2466	0.029*	
H8B	0.1019	1.0668	0.3100	0.029*	
H8C	0.0659	0.8671	0.2970	0.029*	
C9	0.17735 (8)	0.77377 (19)	0.20373 (6)	0.0144 (3)	
C10	0.10569 (8)	0.69513 (19)	0.18460 (6)	0.0165 (3)	
H10	0.0803	0.6507	0.2112	0.020*	
C11	0.07151 (8)	0.68226 (19)	0.12579 (6)	0.0170 (3)	
H11	0.0222	0.6295	0.1123	0.020*	
C12	0.10849 (8)	0.74559 (19)	0.08639 (6)	0.0165 (3)	
C13	0.18234 (8)	0.81476 (19)	0.10713 (6)	0.0158 (3)	
H13	0.2092	0.8524	0.0807	0.019*	
C14	0.21737 (8)	0.82962 (19)	0.16553 (6)	0.0152 (3)	
H14	0.2676	0.8769	0.1792	0.018*	
C15	0.06899 (9)	0.7449 (2)	0.02308 (6)	0.0218 (3)	
H15A	0.0200	0.6814	0.0161	0.033*	
H15B	0.1011	0.6822	0.0026	0.033*	
H15C	0.0602	0.8708	0.0092	0.033*	
C16	0.37098 (8)	0.9940 (2)	0.61179 (6)	0.0176 (3)	
H1	0.3146 (11)	0.898 (3)	0.4877 (9)	0.037 (5)*	

### Atomic displacement parameters (Å^2^)

**Table:**

	*U*^11^	*U*^22^	*U*^33^	*U*^12^	*U*^13^	*U*^23^
S1	0.01806 (18)	0.0235 (2)	0.01600 (19)	−0.00002 (14)	0.00481 (14)	−0.00172 (14)
N1	0.0221 (6)	0.0185 (6)	0.0116 (6)	−0.0013 (5)	0.0040 (5)	−0.0010 (5)
N2	0.0195 (6)	0.0184 (6)	0.0159 (6)	−0.0008 (5)	0.0035 (5)	−0.0004 (5)
N3	0.0133 (5)	0.0157 (6)	0.0141 (6)	−0.0002 (5)	0.0015 (4)	0.0008 (5)
N4	0.0144 (5)	0.0142 (5)	0.0134 (6)	−0.0009 (5)	0.0030 (4)	−0.0001 (5)
N5	0.0310 (7)	0.0304 (7)	0.0158 (7)	−0.0012 (6)	0.0078 (5)	−0.0009 (6)
C1	0.0170 (7)	0.0225 (7)	0.0172 (7)	0.0005 (6)	0.0018 (5)	−0.0019 (6)
C2	0.0171 (7)	0.0142 (7)	0.0143 (7)	−0.0026 (6)	0.0026 (5)	0.0002 (5)
C3	0.0175 (7)	0.0124 (6)	0.0142 (7)	−0.0024 (5)	0.0045 (5)	0.0002 (5)
C4	0.0171 (6)	0.0125 (6)	0.0141 (7)	−0.0016 (5)	0.0040 (5)	−0.0001 (5)
C5	0.0201 (7)	0.0141 (6)	0.0150 (7)	−0.0019 (6)	0.0050 (5)	0.0010 (5)
C6	0.0261 (8)	0.0248 (8)	0.0175 (7)	0.0029 (6)	0.0094 (6)	−0.0016 (6)
C7	0.0184 (7)	0.0125 (6)	0.0136 (7)	−0.0016 (5)	0.0053 (5)	−0.0005 (5)
C8	0.0184 (7)	0.0221 (8)	0.0169 (7)	0.0027 (6)	0.0040 (6)	−0.0007 (6)
C9	0.0173 (6)	0.0138 (6)	0.0112 (6)	0.0015 (5)	0.0024 (5)	0.0003 (5)
C10	0.0182 (7)	0.0166 (7)	0.0152 (7)	−0.0011 (6)	0.0054 (5)	0.0012 (5)
C11	0.0156 (6)	0.0165 (7)	0.0174 (7)	−0.0026 (6)	0.0020 (5)	−0.0013 (6)
C12	0.0189 (7)	0.0153 (7)	0.0146 (7)	0.0025 (6)	0.0036 (5)	−0.0003 (5)
C13	0.0178 (7)	0.0169 (7)	0.0141 (7)	0.0021 (6)	0.0065 (5)	0.0009 (5)
C14	0.0137 (6)	0.0144 (7)	0.0173 (7)	0.0001 (5)	0.0042 (5)	−0.0007 (5)
C15	0.0223 (7)	0.0269 (8)	0.0143 (7)	0.0006 (6)	0.0018 (6)	0.0006 (6)
C16	0.0198 (7)	0.0170 (7)	0.0189 (7)	0.0010 (6)	0.0105 (6)	0.0020 (6)

### Geometric parameters (Å, °)

**Table:**

S1—C16	1.6390 (15)	C6—H6B	0.9800
N1—C5	1.3229 (18)	C6—H6C	0.9800
N1—N2	1.3738 (17)	C7—C8	1.4877 (19)
N1—H1	0.91 (2)	C8—H8A	0.9800
N2—C2	1.3097 (18)	C8—H8B	0.9800
N3—C3	1.3293 (18)	C8—H8C	0.9800
N3—N4	1.3755 (16)	C9—C10	1.386 (2)
N4—C7	1.3533 (17)	C9—C14	1.3905 (19)
N4—C9	1.4320 (17)	C10—C11	1.3915 (19)
N5—C16	1.164 (2)	C10—H10	0.9500
C1—C2	1.4879 (19)	C11—C12	1.393 (2)
C1—H1A	0.9800	C11—H11	0.9500
C1—H1B	0.9800	C12—C13	1.396 (2)
C1—H1C	0.9800	C12—C15	1.5034 (19)
C2—C3	1.4294 (19)	C13—C14	1.3863 (19)
C3—C4	1.4124 (19)	C13—H13	0.9500
C4—C7	1.3984 (19)	C14—H14	0.9500
C4—C5	1.4025 (19)	C15—H15A	0.9800
C5—C6	1.492 (2)	C15—H15B	0.9800
C6—H6A	0.9800	C15—H15C	0.9800
			
C5—N1—N2	127.91 (12)	N4—C7—C8	124.77 (12)
C5—N1—H1	120.8 (12)	C4—C7—C8	130.84 (13)
N2—N1—H1	111.3 (12)	C7—C8—H8A	109.5
C2—N2—N1	117.73 (12)	C7—C8—H8B	109.5
C3—N3—N4	102.82 (11)	H8A—C8—H8B	109.5
C7—N4—N3	114.78 (11)	C7—C8—H8C	109.5
C7—N4—C9	127.13 (12)	H8A—C8—H8C	109.5
N3—N4—C9	118.08 (11)	H8B—C8—H8C	109.5
C2—C1—H1A	109.5	C10—C9—C14	121.39 (13)
C2—C1—H1B	109.5	C10—C9—N4	120.13 (12)
H1A—C1—H1B	109.5	C14—C9—N4	118.48 (12)
C2—C1—H1C	109.5	C9—C10—C11	118.94 (13)
H1A—C1—H1C	109.5	C9—C10—H10	120.5
H1B—C1—H1C	109.5	C11—C10—H10	120.5
N2—C2—C3	119.82 (13)	C12—C11—C10	121.00 (13)
N2—C2—C1	118.53 (12)	C12—C11—H11	119.5
C3—C2—C1	121.65 (13)	C10—C11—H11	119.5
N3—C3—C4	112.43 (12)	C11—C12—C13	118.48 (13)
N3—C3—C2	127.69 (13)	C11—C12—C15	120.84 (13)
C4—C3—C2	119.87 (13)	C13—C12—C15	120.65 (13)
C7—C4—C5	135.66 (13)	C14—C13—C12	121.43 (13)
C7—C4—C3	105.60 (12)	C14—C13—H13	119.3
C5—C4—C3	118.72 (12)	C12—C13—H13	119.3
N1—C5—C4	115.91 (13)	C13—C14—C9	118.58 (13)
N1—C5—C6	118.14 (13)	C13—C14—H14	120.7
C4—C5—C6	125.95 (13)	C9—C14—H14	120.7
C5—C6—H6A	109.5	C12—C15—H15A	109.5
C5—C6—H6B	109.5	C12—C15—H15B	109.5
H6A—C6—H6B	109.5	H15A—C15—H15B	109.5
C5—C6—H6C	109.5	C12—C15—H15C	109.5
H6A—C6—H6C	109.5	H15A—C15—H15C	109.5
H6B—C6—H6C	109.5	H15B—C15—H15C	109.5
N4—C7—C4	104.34 (12)	N5—C16—S1	179.46 (14)
			
C5—N1—N2—C2	−0.3 (2)	C9—N4—C7—C4	179.09 (12)
C3—N3—N4—C7	1.25 (15)	N3—N4—C7—C8	175.84 (12)
C3—N3—N4—C9	−179.52 (11)	C9—N4—C7—C8	−3.3 (2)
N1—N2—C2—C3	−1.58 (19)	C5—C4—C7—N4	−177.34 (15)
N1—N2—C2—C1	177.60 (12)	C3—C4—C7—N4	1.48 (15)
N4—N3—C3—C4	−0.19 (15)	C5—C4—C7—C8	5.3 (3)
N4—N3—C3—C2	178.40 (13)	C3—C4—C7—C8	−175.91 (14)
N2—C2—C3—N3	−176.53 (13)	C7—N4—C9—C10	−48.7 (2)
C1—C2—C3—N3	4.3 (2)	N3—N4—C9—C10	132.23 (13)
N2—C2—C3—C4	2.0 (2)	C7—N4—C9—C14	131.16 (15)
C1—C2—C3—C4	−177.20 (13)	N3—N4—C9—C14	−47.96 (17)
N3—C3—C4—C7	−0.83 (16)	C14—C9—C10—C11	−3.9 (2)
C2—C3—C4—C7	−179.55 (12)	N4—C9—C10—C11	175.90 (12)
N3—C3—C4—C5	178.22 (12)	C9—C10—C11—C12	0.5 (2)
C2—C3—C4—C5	−0.5 (2)	C10—C11—C12—C13	3.0 (2)
N2—N1—C5—C4	1.7 (2)	C10—C11—C12—C15	−175.17 (13)
N2—N1—C5—C6	−178.41 (13)	C11—C12—C13—C14	−3.3 (2)
C7—C4—C5—N1	177.48 (15)	C15—C12—C13—C14	174.88 (13)
C3—C4—C5—N1	−1.22 (19)	C12—C13—C14—C9	0.0 (2)
C7—C4—C5—C6	−2.4 (3)	C10—C9—C14—C13	3.6 (2)
C3—C4—C5—C6	178.94 (13)	N4—C9—C14—C13	−176.18 (12)
N3—N4—C7—C4	−1.76 (16)		

### Hydrogen-bond geometry (Å, °)

**Table:**

*D*—H···*A*	*D*—H	H···*A*	*D*···*A*	*D*—H···*A*
N1—H1···N5	0.91 (2)	1.86 (2)	2.7668 (18)	175.6 (19)
